# Me, My Child, and Us: A Group Parenting Intervention for Parents with Lived Experience of Psychosis

**DOI:** 10.3390/bs15070950

**Published:** 2025-07-14

**Authors:** Nithura Sivarajah, Jessica Radley, Rebecca Knowles-Bevis, Louise C. Johns

**Affiliations:** 1 Department of Psychiatry, University of Oxford, Oxford OX37JX, UK; jessica.radley@kcl.ac.uk (J.R.); louise.johns@psych.ox.ac.uk (L.C.J.); 2 Oxford Health NHS Foundation Trust, Oxford OX44XN, UK; 3 West London NHS Trust, London UB24SD, UK; 4 Kings College London, University of London, London WC2R2LS, UK; 5 Babies 1st CIC, Buckinghamshire HP137TJ, UK

**Keywords:** psychosis, parents, parenting, mentalization, group therapy

## Abstract

Many patients with psychosis have dependent children. Being a parent is an important and valued role for people with psychosis. However, the experience of psychosis can disrupt parent–child interactions, which can negatively affect both parents and children. Despite this understanding, there remains a lack of diagnosis-specific parenting interventions for parents with lived experience of psychosis. An eight-week digital mentalization-based parenting group intervention (Me, My Child, and Us) was piloted to evaluate its acceptability, feasibility, and impact on self-reported parenting satisfaction, parental relationship, and overall wellbeing. The study used a within-participant non-controlled pre–post design using mixed quantitative and qualitative methodology. Thirteen parents with dependent children were recruited and two eight-week groups were run. Eleven parents completed the intervention, the pre- and post-group measures, and provided qualitative feedback on their experience of the intervention. On average, parents attended 75% of sessions. Parents reported high satisfaction with the content and structure of the group. Scores on pre- and post- group measures suggest improvements in self-reported parental wellbeing, parental relationship, parenting stress levels, parenting satisfaction and efficacy, as well as mentalizing capacity. The Me, My Child, and Us parenting group is feasible to deliver and acceptable for parents with lived experience of psychosis. The preliminary self-report data indicate a controlled evaluation of the intervention as the next step.

## 1. Introduction

Living with psychosis can impact social, emotional, and psychological functioning (Booij et al., 2018). Stressful interpersonal relationships and social difficulties have been found to exacerbate psychotic symptoms and have the potential to trigger relapse (e.g., The Stress Vulnerability Model, Wied & Jansen, 2002; Radley et al., 2022c). The exact prevalence of psychosis amongst parents is uncertain, although previous studies have reported that more than 50% of women and 25% of men with psychosis are parents (Campbell et al., 2012; Radley et al., 2022b). Parenting stress has specifically been identified as a potential exacerbator of psychotic symptoms and can impact parents’ levels of distress and day-to-day functioning (Radley et al., 2022a; Wolfenden et al., 2022).

Managing the dual demands of being a parent and coping with psychosis can make parents with psychosis even more vulnerable to stress (Strand & Rudolfsson, 2020). Studies have found greater severity and longer duration of psychosis symptoms to be correlated with greater parenting difficulties (Campbell et al., 2018).

Parents with psychosis are also likely to encounter greater adversity compared to other parents, including lower social and emotional support (Campbell et al., 2018), financial disadvantage (Montgomery et al., 2011), greater social isolation (Seeman, 2002), and increased stigma (Malhotra et al., 2015). Stress incurred by social adversities and relational difficulties can directly impact parental mental health, child–parent relationships, and children’s mental health.

When considering the impact of symptoms of psychosis on parent–child relationships, it is important to recognize the episodic and fluctuating nature of psychosis (Blegen et al., 2012; Krumm et al., 2013). Fluctuations in symptoms can have significant impact on various parenting competencies. Positive symptoms, such as delusional ideas or hallucinations, can affect parents’ ability to provide support and participate in reciprocal interactions (Healy et al., 2016; Strand et al., 2020; Kahl & Jungbauer, 2014). Negative symptoms and side effects of antipsychotic medication can manifest as lethargy, irritability, poor concentration, blunted affect, withdrawal, and lack of motivation (Evenson et al., 2008; Dolman et al., 2013), which can then interfere with parents’ ability to engage emotionally with their children (Montag et al., 2014) as well as disciplining and setting boundaries with them (Campbell et al., 2018). Furthermore, cognitive impairments associated with both positive and negative symptoms of psychosis can make it difficult for parents to plan and achieve day-to-day parenting tasks (Nicholson & Miller, 2008).

Fluctuations in symptoms can especially affect parents’ ability to take their child’s perspective which consequently impacts their capacity to meet their children’s needs (Strand et al., 2020; Grusec & Davidov, 2010). The capacity for parents to separate their own mental states from those of their child and then attune to the child’s mental states plays a central role in responsive and effective parenting (Camoirano, 2017). The capacity to imagine, recognize, and reflect upon one’s own mental state and simultaneously to attune to others’ mental states (including feelings, thoughts, desires, and beliefs) is referred to as mentalization (Fonagy & Target, 2006; Luyten et al., 2017; Sharp & Fonagy, 2008).

Problems with mentalization are common amongst people with psychosis (Sprong et al., 2007; Strand et al., 2020) as stressful situations can trigger lapses in mentalizing (Pereira et al., 2012). When parents experience stress, it becomes harder for them to show parental sensitivity and remain flexible in their thinking. At these times, parents may jump to conclusions about their child’s intentions (Fishburn, 2017) or they may be overly focused on and influenced by their own internal world at the expense of noticing and acknowledging their child’s mental states. Mentalization-based treatment (MBT) aims to improve parents’ ability to respond in a timely and appropriate way to children’s communication by fostering curiosity about the child’s inner world (Sleed et al., 2021).

Evidence suggests that impairments in mentalization and parenting are most pronounced during acute psychotic episodes, when symptoms such as delusions, hallucinations, and disorganized thinking disrupt reflective functioning and emotional regulation (Fonagy & Bateman, 2016). During these periods, parents may struggle to interpret their children’s needs and respond sensitively, affecting parent–child interactions (Sharp & Fonagy, 2008). However, mentalizating and parenting difficulties may not fully remit even during symptom remission. Research indicates that subtle impairments in social cognition, including theory of mind and affective mentalization, can persist between psychotic episodes (Brüne, 2005; Montag et al., 2010). These residual difficulties may contribute to ongoing challenges in parenting, such as difficulties in perspective-taking and emotion regulation, which can affect the quality of parent–child relationships (Pajulo et al., 2015). Consequently, parenting support for this population should address both the acute symptom phases and the more enduring cognitive and emotional challenges beyond acute illness episodes (Reupert et al., 2015).

At the same time, it is equally important to note that the parental role can also foster hope, a sense of purpose, connection and identity (Leamy et al., 2011) which can support individual recovery, improve familial relationships, and support with relational resilience within the family system. Parenting can contribute positively not only to parents’ psychological wellbeing, personal identity, and illness recovery, but also to their children’s mental health and the quality of the parental relationship (Dolman et al., 2013; Reupert et al., 2015; Hinchliffe et al., 2022).

Despite the understanding of the need for support for parents with psychosis, there is a lack of evidence-based, effective, and timely parenting interventions (Radley et al., 2022e; Schrank et al., 2015). Existing programs such as Triple P (Wolfenden et al., 2022) and Family Talk (Strand & Meyersson, 2020) have been trialed with parents who experience psychosis, yet these interventions were originally designed for broader populations and do not directly address the distinctive psychological and relational needs associated with psychosis.

Given the additional challenges associated with psychosis, generalized or universal parenting programmes are likely to be of limited usefulness and may even be detrimental (Strand et al., 2020; Radley et al., 2022d). Tailored interventions are needed (Grusec & Davidov, 2010) to enable parents with psychosis to increase their capacity for mentalization and become better at identifying where symptoms might hinder positive parenting behaviors (Strand et al., 2020).

Qualitative interview studies with parents with psychosis and their family members highlight that parents with psychosis would benefit from (1) peer support from other parents with psychosis, (2) understanding the relationship between psychosis and parenting stress, and (3) learning skills to improve communication between family members (Radley et al., 2022c, 2022d).

Digital group-based interventions may offer a promising avenue for supporting parents with psychosis by addressing both accessibility barriers and the psychosocial needs of this population. Digital platforms enhance flexibility, privacy, and scalability, overcoming logistical challenges such as stigma and social isolation while facilitating tailored, interactive content suitable for cognitive difficulties associated with psychosis (Gillard et al., 2015; Bucci et al., 2018; Rotondi et al., 2010). Concurrently, the group format fosters peer support, normalization, and the development of parenting skills through shared experiences and reciprocal feedback, which are critical for improving reflective functioning and mentalization in parent–child relationships (Reupert et al., 2015; Shaw et al., 2016; Fonagy & Bateman, 2016). Integrating digital delivery with group-based modalities thus combines the benefits of increased accessibility and psychosocial support, representing a scalable, cost-effective approach to meeting the unique and complex needs of parents with psychosis (Mohr et al., 2013; Gillard et al., 2015).

Me, My Child, and Us, an eight-week digital mentalization-based group intervention tailored specifically for parents with psychosis with children aged 2–18 years old, was co-developed with experts by experience and offered to support parents with psychosis through addressing the needs described above. The primary aim of the study was to ascertain the acceptability and feasibility of the intervention for parents with psychosis in real-world clinical settings: adult community mental health teams. A secondary aim was to investigate whether there was any change in parents’ stress levels, mental health, and parent–child relationships following engagement in the group.

## 2. Materials and Methods

### 2.1. Design

The project received quality improvement approval by the host National Health Service (NHS) Foundation Trust. All participating parents provided written consent for their data to be included in the project.

A within-participant non-controlled pre–post design was employed. A convergent parallel design was used, where quantitative and qualitative data were collected concurrently, analyzed separately, and then merged for interpretation and evaluation concerning the acceptability, feasibility, and perceived change following completing the intervention.

### 2.2. Participants

Parents were invited to take part in the group if they met the following criteria: (1) English speaking, (2) reported experiences of psychosis supplemented by diagnosis of schizophrenia and schizoaffective disorder from the ICD-11 (active positive symptoms were not an exclusion criteria), (3) at least 18 years of age, and (4) being a parent of a child aged 2–18 years old (level of contact was not an eligibility criterion). Recruitment occurred through liaison with keyworkers in community mental health teams serving people with early (e.g., first episode) and established psychosis. Forty-one referrals were received, and twenty-eight people expressed an interest in the group. Fifteen were either unable to commit to the time of the intervention or could not be contacted before the start of the group. Thirteen parents provided written consent and took part in the intervention. Two parents dropped out: one due to ongoing court proceedings and the other due to physical ill health. See Table 1 for an overview of participant characteristics.

### 2.3. Measures

Feasibility and acceptability were assessed by session attendance, satisfaction ratings, and participant feedback from a feedback questionnaire, which focused on the value and usefulness of the intervention and delivery format. The feedback questionnaire consisted of 22 questions. The first 17 were questions about the intervention, delivery, and format, and it used a Likert rating scales (satisfied to dissatisfied, very helpful to very unhelpful, and agree to disagree) and the remaining 5 were open-ended qualitative questions (e.g., tell us about any changes you noticed in yourself, your parenting skills or in your relationship with your child/children?).

Outcome measures included standardized and validated self-report questionnaires assessing mood, parenting, parental stress, and parent–child relationships (see Table 2). Measures were completed online using Microsoft Forms prior to the first session and immediately after the final (eighth) session.

### 2.4. Procedure

Two rounds of the intervention (Me, My Child, and Us) were facilitated, the first between January and March 2022 and the second during October and December 2022. Prior to starting the intervention, one of the group facilitators contacted each participant and administered the baseline measures with them via Microsoft Forms. In line with other existing parenting programs, the intervention comprised of eight weekly sessions. Each session lasted for 90 min with a 10 min break halfway through.

A semi-manualized PowerPoint presentation was developed to structure and guide the group sessions, and a client workbook was developed summarizing the content of the PowerPoint presentation. Each participant received a copy of the workbook to use both during the sessions and as a resource for continued engagement between sessions.

Principles of mentalization underpinned the intervention. Each session followed the same structure: a general check-in with the group, homework review, planning the session ahead, and the agenda topic. The sessions concluded with homework setting and a brief guided relaxation or mindfulness exercises. The contents included didactic psychoeducational elements, video clips, experiential exercises, group discussions, self-reflective exercises, and homework setting. See Table 3 for an overview of the intervention.

The intervention was facilitated by a final year trainee clinical psychologist, a consultant clinical psychologist, and a final year PhD student/assistant psychologist. Facilitators modelled key skills, provided illustrative examples, and played scripted videos to demonstrate new skills and strategies. Experiential exercises were brief and scaffolded, and learning points were summarized to accommodate for everyone’s information processing speed and emotional arousal. Participants were invited to practice the new skills on their own and with each other in small groups. This active, experiential element was included to consolidate participants’ learning, normalize their experiences- and promote peer support in a non-judgmental way.

Each participant was contacted by a facilitator once every week between sessions for a 10–20 min check-in call. These calls allowed parents to share their learning, provide feedback on the previous session, discuss individual experiences relating to the session content, and share their learning from the homework.

After completing the intervention, all participants were asked to complete the battery of outcome measures a second time on Microsoft Forms.

To ensure adherence with the intervention, facilitators followed a detailed session-by-session semi-scripted protocol guided by the notes section on the PowerPoint presentation. All eight sessions were led by the first author with support from the remaining authors. All facilitators attended a brief pre- and post-intervention meeting each week. For the first group, all facilitators met weekly for a 60 min peer supervision session to plan the upcoming session, discuss reflexivity and problem solve concerns and queries.

Following completion of the group, participants were invited to complete a feedback questionnaire (see Supplementary Materials).

### 2.5. Data Analysis

In line with a convergent parallel mixed methods design, quantitative and qualitative data were collected separately, analyzed separately, then integrated for interpretation to provide a comprehensive understanding of the study findings.

Quantitative data were anonymized and analyzed using SPSS version 25.0. Descriptive statistics, graphical representations, and mean scores on outcome measures were used to examine patterns in attendance and to ascertain changes following the intervention. Qualitative data from free-text responses on the feedback questionnaires were summarized using a keyword-based approach (Firoozeh et al., 2020), identifying common terms and phrases that reflected participants’ views on the acceptability, feasibility, and perceived effectiveness of the group.

## 3. Results

### 3.1. Acceptability and Feasibility of Me, My Child, and Us

Thirteen parents (eight in the first group and five in the second) commenced the intervention. Two parents dropped out, one from each group. The first participant dropped out in the second session due to an ongoing court proceeding and a second participant in the second group also dropped out in the second session due to physical ill health. Participants were majority female (11 females, 2 male), the average age was 41 (range 24–51). The average number of children per participant was two (range 1–4), children’s ages ranged from 2 to 17 with an average age of 8. Six participants were employed and seven participants were unemployed. Four participants were people of global majority and the remainder self-identified as White British. On average, participants attended and actively engaged in six sessions, with attendance ranging from two to eight sessions. In total, 31% of participants attended all eight sessions, 15.3% attended seven sessions, 23% attended six sessions, 15.3% attended five sessions, and 15.3% attended two sessions. All participants engaged in individual check-in calls following each session they attended. No adverse events were reported throughout the groups.

**Figure 1:**
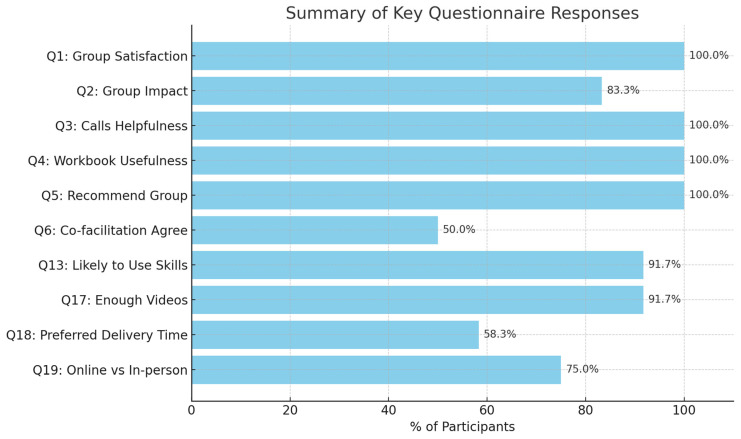
This figure presents the most frequent responses to selected questions from the post-group feedback questionnaire (N = 12). Each bar displays the percentage of participants endorsing the most common answer. The findings indicate high levels of satisfaction, perceived benefit, and intent to apply parenting strategies learned during the intervention. Participants also favored the online format and found the group content and structure appropriate and helpful.

Descriptive statistics were used to analyze the scores from the first section of the feedback questionnaire (quantitative data). All participants (100%) rated being *satisfied* with the group. Ten (83.3%) rated that the intervention had *made things better*, with the remaining two (16.7%) rating it as having *made things slightly better*. All participants (100%) rated the between-session calls and the workbook as *very helpful*. All participants (100%) rated *definitely yes* when asked if they would recommend the group to others. When asked, “Do you think you are likely to use the parenting skills and techniques?”, 11 (91.7%) rated *definitely yes*, and the remaining parent (*n* = 1; 8.3%) rated, *possibly yes*. Twelve participants provided additional qualitative feedback. Figure 1 and Table 4 summarizes the rating and qualitative results of the feedback questionnaire.

Parents’ open-ended feedback included that they became better at mentalizing, better listeners, felt calmer in themselves, and gained confidence in their role as parents. See Table 4 for a summary of participant feedback and see Supplementary Materials for a comprehensive overview of the participant feedback.

### 3.2. Effectiveness of Me, My Child, and Us

A complete case analysis from the eleven participants who completed the intervention were analyzed. Data from the two participants who dropped out were omitted due to the absence of post-intervention data. Wilcoxon Signed-Rank Tests were used to analyze pre- and post-intervention scores for all questionnaires. There were statistically significant improvements on all outcome measures following the group, except for the Dependence Subscale on the Child Parent Relationship Scale. See Table 5 for a summary of changes in mean scores pre- and post-intervention. Detailed plot diagrams illustrating individual and group mean pre- and post-intervention scores for each questionnaire are provided in Supplementary Materials.

## 4. Discussion

The primary aim of the study was to assess the acceptability and feasibility of an eight-week digital mentalization-based group intervention (Me, My Child, and Us), tailored specially for parents with psychosis. The qualitative and quantitative findings were complementary, and both reinforced the intervention’s promising acceptability, feasibility, and preliminary effectiveness, suggesting that the group was well received, perceived as beneficial, and would be feasible to implement in community mental health services. Quantitative results demonstrated promising indicators across all domains, including favorable completion rates, participant retention, and improvements in key outcome measures. Participants reported improvement in general wellbeing, parental stress levels, parenting efficacy and satisfaction, parental relationships, and mentalizing capacity. These findings were substantiated by qualitative data, which provided contextualized insights into participants’ experiences of the intervention. Specifically, participants’ reflections on the intervention’s relevance, accessibility, and perceived impact helped to elucidate the mechanisms underlying the quantitative trends and offered perspectives beyond what the quantitative data captured alone.

Recruitment rates were good; however, some difficulties were encountered. Keyworkers did not always inform parents on their caseloads about the group, and there were also inconsistencies in recording dependent children on electronic patient records.

Thirteen parents started the intervention across two groups, and eleven (84.6%) completed it. On average, parents attended 75% of sessions, suggesting that the group is feasible for this population. Due to parents’ work commitments, a decision was made to run two groups at different times. The first group occurred between January and March on Mondays between 10.00 a.m. and 11.30 a.m. and the second group was facilitated on Wednesday evenings 6.00 p.m.–07.30 p.m. between October and December. Overall, there was higher attendance in the first group. This might suggest that the time of day and year should be carefully considered when setting up the group, and special consideration may need to be made regarding school holidays.

Both groups were conducted using a digital format (Microsoft Teams). This digital delivery facilitated attendance, allowing parents to join from a wide geographical area, eliminating travel costs and time, reducing the need for childcare, and being able to attend from the comfort of one’s own home or, in some instances, attending whilst at work. From the participant feedback, eleven out of twelve participants (91.7%) stated that given a choice, they would prefer to attend the group online rather than face-to-face. Digital groups could also appeal to parents as they may help to overcome some of the symptom-related and social barriers associated with psychosis (such as difficulties with motivation, social anxiety, financial problems, and disorganization), which might make attendance in the community more difficult. However, there is potential for a biased response regarding preference for digital interventions from our sample, since they attended the digital group. It is likely that a population of parents with psychosis who may not have had high digital literacy were not reached as they were not able to attend in the first place (Watson et al., 2022). The format of Me, My Child, and Us can, however, easily be adapted and delivered face to face if desired.

Reviews of the between-session feedback and feedback questionnaire suggest that beneficial aspects of the Me, My Child, and Us were psychoeducation about psychosis and its impact on parenting, practicing mentalizing and attunement skills, learning communication strategies, and learning skills to manage symptoms of psychosis, as well as peer support and discussions.

To our knowledge, there are 34 empirically researched interventions designed to support parents with a mental illness that would be appropriate for parents with psychosis (Radley et al., 2022e). However, only two of those interventions are specifically designed to focus solely on supporting the parents with psychosis, Triple P (Wolfenden et al., 2022) and Family Talk (Strand & Meyersson, 2020). The remaining parenting interventions primarily focus on supporting the children of parents with psychosis or have adopted a broader family-based approach involving families in which at least one parent experiences psychosis (e.g., CHIMPS; Think Family Whole Family Programme; VIA Family, Young SMILES, SEEK, etc.). Many of these studies also recruited parents with a severe mental illness, not exclusively parents including a diagnosis of schizophrenia, bipolar disorder, or depression.

Neither the Triple P intervention nor the Family Talk intervention are adapted to the specific needs of parents with psychosis nor are they based on the principles of mentalization. This is the first intervention that is specifically tailored to the needs of parents with psychosis. Previous studies with parents with psychosis have reported that parents can find it difficult to maintain learning obtained through educational interventions. By incorporating experiential practice in all sessions, setting and reviewing homework, and making support calls between sessions, facilitators supported participants in strengthening and tailoring the skills and techniques to individual parents’ contexts and needs. The workbook also allowed parents to revisit their learning and refresh their memory.

Researchers have described the importance of incorporating parenting support interventions into adult mental health services (Reupert & Maybery, 2009). Whilst some studies have found that clinicians are open to this, other have found that clinicians feel powerless to help service users when they do not have shared experiences (McMullan et al., 2018) or feel ill-equipped to work effectively with needs specifically related to parenting (Radley et al., 2021). Thus, there is a need to support mental health staff to feel more confident in working with patients who are managing the dual demands of psychosis and parenting.

A manualized intervention such as Me, My Child, and Us, which draws on general therapeutic skills and techniques already familiar within adult mental health care (e.g., psychoeducation, mentalization-based approaches, and group facilitation), may offer a scalable and pragmatic solution to this identified gap. The structured format of the group, along with the flexibility to be delivered as either a stand-alone intervention or adjunctive to ongoing psychological care, supports its potential integration into routine service delivery within community mental health teams. The use of semi-manualized materials and a co-facilitated group model also allows for adaptability across different service contexts and levels of staff expertise, which may further facilitate uptake and implementation.

However, potential barriers to wider implementation must also be acknowledged. These include workforce capacity, competing service priorities, and variability in staff confidence or perceived role boundaries in relation to parenting work. Addressing these challenges may require additional training and supervision for facilitators, as well as strategic support from service managers to embed parenting-focused interventions within existing care pathways.

### 4.1. Limitations

Several limitations of this study should be acknowledged, particularly in relation to the generalizability of the findings. First, many of the participants previously took part in an earlier qualitative interview study conducted by one of the authors, during which they described the kinds of support they would find helpful. As a result, the intervention may have been unintentionally tailored to the specific needs of this subgroup of parents, potentially limiting the applicability of the findings to broader populations of parents with psychosis. Additionally, the sample size was small and demographically homogenous, comprising a predominantly white, female participant group. Only two participants were male and fathers, and only two were people of global majority. These demographic limitations restrict the extent to which findings can be generalized across more diverse populations, including fathers, people of global majority, and those with varying cultural understandings of parenting and mental health. Future studies should aim to recruit more diverse samples to explore the acceptability and relevance of the intervention across different demographic contexts.

The absence of a control group also limits the ability to draw conclusions about the specific effects of the intervention, as improvements observed could be attributable to nonspecific factors such as increased attention from clinicians or participation in a supportive group environment. Additionally, no psychosis-specific outcome measures were included. Given established links between parenting stress and psychotic symptomatology, future evaluations should incorporate measures assessing changes in symptom severity, relapse rates, or functional outcomes to better understand the intervention’s potential clinical impact.

The questions and scales included in the feedback questionnaire were developed based on the study objectives with the aim of eliciting the most informative and formative responses from participants. Although consistency across all questions would have been ideal, the focus was not on measuring change but rather on capturing participants’ perceptions and experiences. The limitation of using non-standardized feedback scales is to be acknowledged, and adapting an existing validated satisfaction scale could enhance the rigor of future research. This is an important consideration for a larger subsequent study.

Although no adverse effects were reported for participants, the study did not gather input from co-parents or partners, nor did it assess potential adverse effects for partners or other family members. Moreover, the absence of child perspectives is a notable limitation. With greater time, resources, and ethical approvals, it would have been valuable to include child-reported outcomes to assess changes in the parent–child relationship from the children’s point of view.

Regarding feasibility and implementation, the study was conducted as part of a clinical quality improvement (QI) initiative, with elements of recruitment and delivery led by the research team. Although staff were regularly encouraged to refer suitable clients, systemic barriers were identified. In particular, clinicians often lacked access to accurate and up-to-date information about service users’ parental status or details of dependent children, as these are not always reliably recorded in electronic health records. This limited the pool of eligible participants, also reflects a broader structural issue that could hinder wider implementation of parenting-focused interventions in routine adult community mental health services. These findings highlight the need for services to improve documentation and awareness of parental status, as well as to support staff in identifying and referring parents who may benefit from such interventions.

### 4.2. Future Directions

This project indicated that a mentalization-based parenting group intervention for parents with psychosis is both feasible and acceptable, and it may also be clinically effective. Building on these findings, future research will involve a randomized controlled trial (RCT) designed to evaluate the efficacy of the intervention with a larger and more diverse sample. The RCT will also aim to assess broader outcomes, including parent and child functioning, service use, and implementation metrics, to inform its potential for widespread adoption across early intervention and broader adult mental health services.

## Figures and Tables

**Table 1:** Participant characteristics.

Gender	Age	Ethnicity	No. of Children	Children’s Ages	Number of Sessions Attended
Female	36–45	PoGM	1	6	2
Female	36–45	PoGM	1	3	8
Female	>45	White British	1	15	8
Female	>45	White British	1	10	7
Female	36–45	White British	3	11, 9, 3	8
Female	>45	White British	1	5	6
Female	>45	White British	2	8, 12	7
Female	36–45	White British	4	8, 12, 15, 17	8
Male	26–35	White British	4	8, 5, 2, 3	6
Female	26–35	PoGM	3	13, 11, 8	6
Male	>50	White British	2	16, 4	2
Female	26–35	White British	2	6, 5	5
Female	36–45	PoGM	1	5	5

People of Global Majority (PoGM) included people of Black British, British Polish, British Afghan, and British Pakistani ethnicities.

**Table 2:** Summary of secondary outcome measures and their properties.

Name of Measure	Function	Properties
Clinical Outcomes in Routine Evaluation, CORE-10, (Barkham et al., 2013)	Assesses distress and functioning.	Ten-item questionnaire. Scores are presented as a total score of 0–40 and a mean score of 0–4. Higher scores indicate higher levels of general psychological distress. A total score of 11 or above indicates clinical significance. It has an internal reliability (alpha) of 0.9
Being a Parent Scale, BAPS, (Gibaud-Wallson & Wandersmith, 1978 as cited in Johnston & Mash, 1989)	Assesses parental self-efficacy and satisfaction regarding their parenting experiences.	Sixteen-item questionnaire with a 6-point Likert-type scale, which parents use to answer the extent to which they agree or disagree with statements about their parenting experiences. Scores range from 16 to 96. Scores are divided into satisfaction and efficacy, with lower scores indicating higher efficacy and satisfaction. Cronbach’s alpha coefficients of 0.75 for satisfaction scale and 0.76 for efficacy scale are reported for reliability.
Child–parent Relationship Scale (Pianta, 1992)	Assesses parent–child relationship.	Fifteen-item questionnaire with a 5-point Likert scale. Each item is made up of 2 statements (30 statements in total), reflecting both positive and negative aspects of parent–child relationships. Ratings are clustered into two groups conflict and closeness. Lower scores on the conflict questions indicate more positive experiences, and higher scores for closeness questions indicate more positive experiences. Studies typically find good internal consistency, with Cronbach’s alpha values typically ranging from 0.70 to 0.90 for both the Closeness and Conflict subscales.
The Parental Reflective Functioning Questionnaire, PRFQ (Luyten et al., 2017)	Assesses parents’ capacity to mentalize about and reflect on their actual and evolving relationship with their child(ren), including the parent’s understanding, curiosity about or disavowal of mental states, and the relationship between mental states and behavior.	Eighteen-item questionnaire rated on a 7-point Likert scale. The questionnaire consists of three subscales. Scores range from 18 to 126. There are three subscales, each consist of 6 items and are rated on a 7-point Likert scale. The PRFQ has good internal consistency (Cronbach’s alpha of 0.688, 0.772 and 0.745 per respective subscale). For the pre-mentalizing subscale, higher scores indicate lower levels of parental reflective functioning. For the certainty and interest/curiosity subscales, average scores are considered optimal.
Parental Stress Scale (Berry & Jones, 1995)	Assesses parents’ stress levels associated with child-rearing. It focuses on parents’ perceptions of their parental role rather than on the sources of stress themselves.	An 18-item measure. Scores range from 18 to 90, where higher scores indicate higher stress levels. Acceptable reliability ratings are reported cross-culturally (Nærde & Sommer Hukkelberg, 2020).

**Table 3:** Overview of the Session Content of Me, My Child, and Us.

Session Number	Topic Discussed
One Two	Background to Parenting (as seen in Family Talk, Let’s Talk about children, CHIMPS, Kids Time, Triple P, Beardslee’s preventive family intervention and VIA family): understanding psychosis and the relationship between psychosis and parenting. Mentalization (as seen in the Lighthouse Parenting Programme and Turning into kids): improving mentalization (reflective functioning) and gaining a better understanding of children’s needs.
Three	Attunement (as seen in the Lighthouse Parenting Programme and Turning into kids): learning to better engage with children and learning to better respond to children.
Four	Communication (as seen in Beardslee’s preventive family intervention, Let’s talk about children, Kids Time, Family Talk and Think Family Whole Family Programme): assertiveness techniques and using encouragement, praise, and rewards.
Five	Managing Conflict and Setting Boundaries (as seen in Think Family Whole Family, Triple P and the Parenting Internet Intervention).
Six	Managing Symptoms of Psychosis (components of parental wellbeing or self-care is included in interventions such as CHIMPS Intervention): understanding symptoms of psychosis and learning coping strategies.
Seven	Communication about Psychosis with Children (as seen in Behavioral Family Therapy, Family Talk, CHIMPS Intervention, Child Talks, Kids Time, Triple P, and CHIMPS): myth busting fears about talking to children about psychosis, exploring what language to use and how to be age appropriate when speaking to children about psychosis, and considering what resources to use when talking to children about psychosis.
Eight	Planning for the Future: reflecting on learning, consolidating learning, and planning on how to maintain the use of newly learned skills and techniques.

**Table 4:** Thematic Summary of Participant Feedback (Qualitative Data).

Theme	Description	Illustrative Quotes
Positive parenting changes	Increased attentiveness, improved emotional regulation, and strengthened relationships with children.	“I listen to them more… they seem to want to talk to me more.”
		“I have become more assertive… able to set boundaries better.
Enhanced Understanding and Confidence	Greater self-awareness and confidence as a parent; application of parenting techniques.	“I am a lot more aware of my own emotions now… this helps me to mentalize my children better.”
Social Connection & Empowerment	Feeling supported and less isolated; benefits of sharing experiences with peers.	“It felt really empowering… to hear about similar experiences.”
		“Being in a group with other parents made me feel less lonely.”
Course Design & Delivery	Appreciation of structure, balance, and content (videos, discussion, relaxation, etc.).	“It was a really good course… I wouldn’t change anything.”
Suggestions for expansion	Requests for similar groups focusing on psychosis alone; desire for broader access.	“I would like GPs and social services to be aware of this course.”
Overall Experience	Highly positive reflections; described as transformative and valuable.	“It’s been the best group I have been to.”
		“Perfect intervention for me.”

**Table 5:** Mean scores before and after intervention (N = 11).

Outcome	Pre-Test	Post-Test	Post–Pre	Post-/Pre-Change	*t*-Value	Wilcoxon Signed-Rank Test *p*-Value
	Mean (SE)	Mean (SE)	Mean	%		
**CORE-10 (psychological distress scores)**	14.09 (2.97)	2.82 (1.53)	−11.27	−80.00	4.70	0.001
**Being a Parent Scale**						
Satisfaction Scale	31.09 (1.95)	45.27 (2.63)	14.18	45.61	−7.05	0.001
Efficacy Scale	28.00 (2.05)	38.45 (0.99)	10.45	37.34	−6.52	0.001
**Child–parent Relationship Scale**						
Conflicts Scores	34.27 (3.48)	16.55 (1.20)	−17.73	−51.72	6.56	0.001
Positive aspects of relationship (closeness)	39.55 (1.73)	45.82 (1.01)	6.27	15.86	−4.28	0.001
Dependence Scores	10.18 (0.69)	8.91 (0.49)	−1.27	−12.50	2.01	0.1055
**The parental reflective functioning scale**						
Pre-Mentalizing Modes	2.94 (0.42)	1.09 (0.08)	−1.85	−62.89	4.38	0.001
Certainty about Mental States	3.39 (0.47)	5.65 (0.14)	2.26	66.52	−5.23	0.0015
Interest and Curiosity in Mental States	5.30 (0.47)	7.00 (0.00)	1.70	32.00	−3.64	0.001
**Parental Stress Scale**	50.82 (3.54)	23.64 (1.52)	−27.18	−53.49	8.24	0.001

## Data Availability

The original contributions presented in this study are included in the article and Supplementary Materials. Further inquiries can be directed to the corresponding author.

## Supplementary Materials

The following supporting information can be downloaded at: https://www.mdpi.com/article/10.3390/bs15070950/s1, Conceptualisation of the group, Feedback Questionnaire, Summary of Participant Feedback and Plotted Diagrams of individual pre- and post- questionnaire scores.
