# Supplementary material for: Me, My Child, and Us: A Group Parenting Intervention for Parents with Lived Experience of Psychosis
Source: Behav Sci (Basel). 2025 Jul 14;15(7):950. doi: 10.3390/bs15070950 (PMC12292144; doi:10.3390/bs15070950)
Supplement: Supplementary file 1 [file behavsci-15-00950-s001.zip › behavsci-3673482-supplementary.pdf]

SUPPLEMENTARY MATERIALS

Conceptualisation of the group

In preparation for the development of the intervention, an extensive scoping review was undertaken to identify and explore content of existing parenting interventions for parents with psychosis (Radley et al., 2022d). The first author attended workshops, webinars and online trainings on a range of relevant topics such as mentalization, attunement and attachment in parent-child relationships. The first author also conducted semi-structured interviews with field experts working with psychosis-, communication in psychosis- and parenting-, and two experts with lived experience to learn about (1) care that is currently available in National Health Service routine practice, (2) barriers faced by services to support parents with psychosis, (3) barriers faced by parents with psychosis when seeking support, (4) the nature of support that parents with psychosis want to access, (5) current recommendations made by researchers in the field, and (6) the process in which existing recommendations could effectively translate into a group based intervention. The two experts by experience also contributed to the development of the intervention, including providing feedback on the information sheets, consent forms, and content-, structure, and design of the intervention and respective materials.

The data obtained from the scoping review, training sessions and expert interviews were analyzed using the principles of Reflexive Thematic Analysis (RTA; Braun & Clarke, 2019). This approach was chosen for its emphasis on researcher subjectivity as a resource and its suitability for examining patterns of meaning across qualitative datasets. The analysis was iterative and inductive, involving familiarization with the data, initial coding, theme development, and ongoing refinement of themes to ensure coherence and depth. Two authors collaboratively conducted this phase of analysis, engaging in regular discussions to reflect on emerging themes, resolve interpretative discrepancies, and ensure rigor through reflexive dialogue. The insights generated from this analytic process informed both the structure and content of the intervention, ensuring alignment with the aims of the study.

Interviews with experts by experience and field experts, findings from literature reviews, alongside learning from learning from literature reviews and trainings, were analyzed by the first author using principles of RTA. The codes and themes were discussed and checked iteratively with the second author during their development.

The data obtained from the scoping review, training sessions and expert interviews were analyzed using the principles of Reflexive Thematic Analysis (RTA; Braun & Clarke, 2019). This approach was chosen for its emphasis on researcher subjectivity as a resource and its suitability for examining patterns of meaning across qualitative datasets. The analysis was iterative and inductive, involving familiarization with the data, initial coding, theme development, and ongoing refinement of themes to ensure coherence and depth. Two authors collaboratively conducted this phase of analysis, engaging in regular discussions to reflect on emerging themes, resolve interpretative discrepancies, and ensure rigor through reflexive dialogue. The insights generated from this analytic process informed both the structure and content of the intervention, ensuring alignment with the aims of the study.

Feedback Questionnaire

Me, My Child & Us Feedback Questionnaire

Getting feedback on your overall experience of the group is extremely important to us. Please complete the below questions to tell us about your experience of the Me, My Child & Us parenting group.

1. How did you find the parenting group?
   Satisfied
   Slightly Satisfied
   Unsure
   Slightly Dissatisfied
   Dissatisfied

2. How did the group impact you?
   Made things better
   Made things slightly better
   No change
   Made things slightly worse
   Made things worse

3. How did you find the in-between session calls?
   Very helpful
   Slightly helpful
   Unsure
   Slightly unhelpful
   Very unhelpful

4. How did you find the workbook?
   Very helpful
   Slightly helpful
   Unsure
   Slightly unhelpful
   Very unhelpful

5. Would you recommend the group to others?
   Definitely yes
   Possibly yes
   Unsure
   Possibly no
   Definitely no

6. The group would be improved if it were co-facilitated by a parent with lived experience of psychosis, e.g., a parent with psychosis delivered some of the material.
   Agree
   Slightly agree
   Unsure
   Slightly disagree
   Disagree

7. Was there enough focus on parenting skills and techniques (e.g., Attunement, mentalising, communication, setting boundaries, STOPP and problem-solving)?
   Not enough
   Just right
   Too much

8. Was there enough focus on managing psychosis?
   Not enough
   Just right
   Too much

9. Was there enough focus on the impact of psychosis on you and your children?
   Not enough
   Just right
   Too much

10. Was there enough time to talk about your experience of psychosis?
    Not enough
    Just right
    Too much

11. Were eight sessions enough?
    Not enough
    Just right
    Too much

12. Was 90mins the right length of time for each session?
    Too short
    Just right
    Too long

13. Do you think you are likely to use parenting skills and techniques?
    Definitely not
    Possibly not
    Unsure
    Possibly yes
    Definitely yes

14. Was there enough time to discuss in small groups/breakout rooms?
    Not enough
    Just right
    Too much

15. Was there enough time to discuss in the big group?
    Not enough
    Just right
    Too much

16. Was there enough information delivered by the facilitators in the sessions?
    Not enough
    Just right

Too much

17. Were there enough videos?
    Not enough
    Just right
    Too much

18. Tell us about any changes you have noticed in yourself, parenting skills, or your relationship with your child(ren).

19. What improvement would you make to the group (is there anything you would change, add or remove)?

20. The early intervention service is available for up to three years. At what stage in an individual's care should this group be delivered? E.g., within the first six months/the last year.

21. Given a choice, would you have preferred the group to be online or in person?

22. Any other comments

Summary of Participant Feedback

Summary of Participant Feedback (N=12)

Feedback	N	%
**1. How did you find the parenting group?**		
Satisfied	12	100
**2. How did the group impact you?**		
Made things better	10	83.33
Made things slightly better	2	16.67
**3. How did you find the in-between session calls?**		
Very helpful	12	100
**4. How did you find the workbook?**		
Very helpful	12	100
**5. Would you recommend the group to others?**		
Definitely yes	12	100
**6. The group would be improved if it were co-facilitated by a parent with lived experience of psychosis, e.g., a parent with psychosis delivered some of the material.**		
Agree	6	50
Slightly agree	2	16.67
Unsure	4	33.33
**7. Was there enough focus on parenting skills and techniques (e.g., Attunement, mentalising, communication, setting boundaries, STOPP and problem-solving)?**		
Just right	12	100
**8. Was there enough focus on managing psychosis?**		
Just right	11	91.67
Not enough	1	14.29
**9. Was there enough focus on the impact of psychosis on you and your children?**		
Just right	12	100
**10. Was there enough time to talk about your experiences of psychosis?**		
Just right	12	100
**11. Were eight sessions enough?**		
Just right	12	100
**12. Was 90 mins the right length of time for each session?**		
Just right	12	100
**13. Do you think you are likely to use parenting skills and techniques?**		
Definitely yes	11	91.67
Possibly yes	1	14.29
**14. Was there enough time for discussions in small groups/breakout rooms?**		

**Feedback**	**N**	**%**
Just right	12	100
**15. Was there enough time to discuss in the big group?**		
Just right	12	100
**16. Was there enough information delivered by the facilitators in the sessions?**		
Just right	12	100
**17. Were there enough videos?**		
Just right	11	91.67
Not enough	1	14.29
**18. The early intervention service is available for up to three years. At what stage in an individual's care should this group be delivered? E.g., within the first six months/the last year.**		
First six months	7	58.33
After one year	1	8.33
Depends on how ill the parent is. For me, it would have been good in the first six months because parenting is a big part of my goals	2	16.67
Not sure	2	16.67
**19. Given a choice, would you have preferred the group to be online or in person?**		
Doesn't matter whether it is online or face to face	2	16.66
In-person	1	8.33
Online	9	75
**20. Tell us about any changes you have noticed in yourself, parenting skills, or your relationship with your child(ren)?**		
• Definitely, yes, I listen to them more, and I pay more attention to them, which means that they seem to want to talk to me a bit more and spend more time with me. This is making me more confident as a parent. Spending more time with the kids has also helped with managing the voices.<br>• I am listening to my children more. I am paying more attention to their needs, understanding them better, and spending more time interacting, engaging, and playing with them. They are sharing more information with me. We have fewer arguments. I have become more assertive and have been able to set boundaries better.<br>• I have learnt to better understand my daughter, she has some developmental delays, but I understand that being patient and tuning into her inner world by mentalising helps me better understand her and respond better to her needs.<br>• I used to feel frustrated before, but as a parent, I understand that I need to take the initiative to repair ruptures. I have noticed that I am more relaxed than before. I am a lot more aware of my own emotions now. This helps me to mentalize my children better. My children are now also more patient with me because I am more open with them. We also have clearer boundaries.		
**21. What improvements would you make to the group (is there anything you would change, add, or remove)?**		

Feedback	N	%
• The course was really well-balanced. There was a good variety of material, teaching, videos, discussions, homework, and relaxation. I've never been to any groups before or even courses on psychosis, so it felt really empowering to know that I was not alone and to be able to share and hear about similar experiences. I wouldn't change anything. This probably wasn't the place, but if there were any similar groups just focusing on and understanding psychosis, I would like to go to that.<br>• Even though I did not talk much, it would have been nice to hear more about people's stories of their psychosis.		
**22. Any other comments:**		
• It was a really good course; I enjoyed the format. It was organised really well, and I would love to be involved in anything else that you might do in the future. Thank you.<br>• It was the perfect intervention for me. I would like GPs and social services to be aware of this course - it would be great for this to be available across the country. It would really help people. I can see a huge difference in myself, my psychosis, my relationship with my children and their well-being.<br>• It’s been really helpful. Now it’s just about keeping on doing what I am doing - and I have the workbook I can go back to if I forget anything.<br>• It’s been the best group I have been to. I have taken away so much in a small amount of time. Being in a group with other parents also made me feel less lonely and more normal.		

Plotted diagrams of individual pre- and post- questionnaire scores

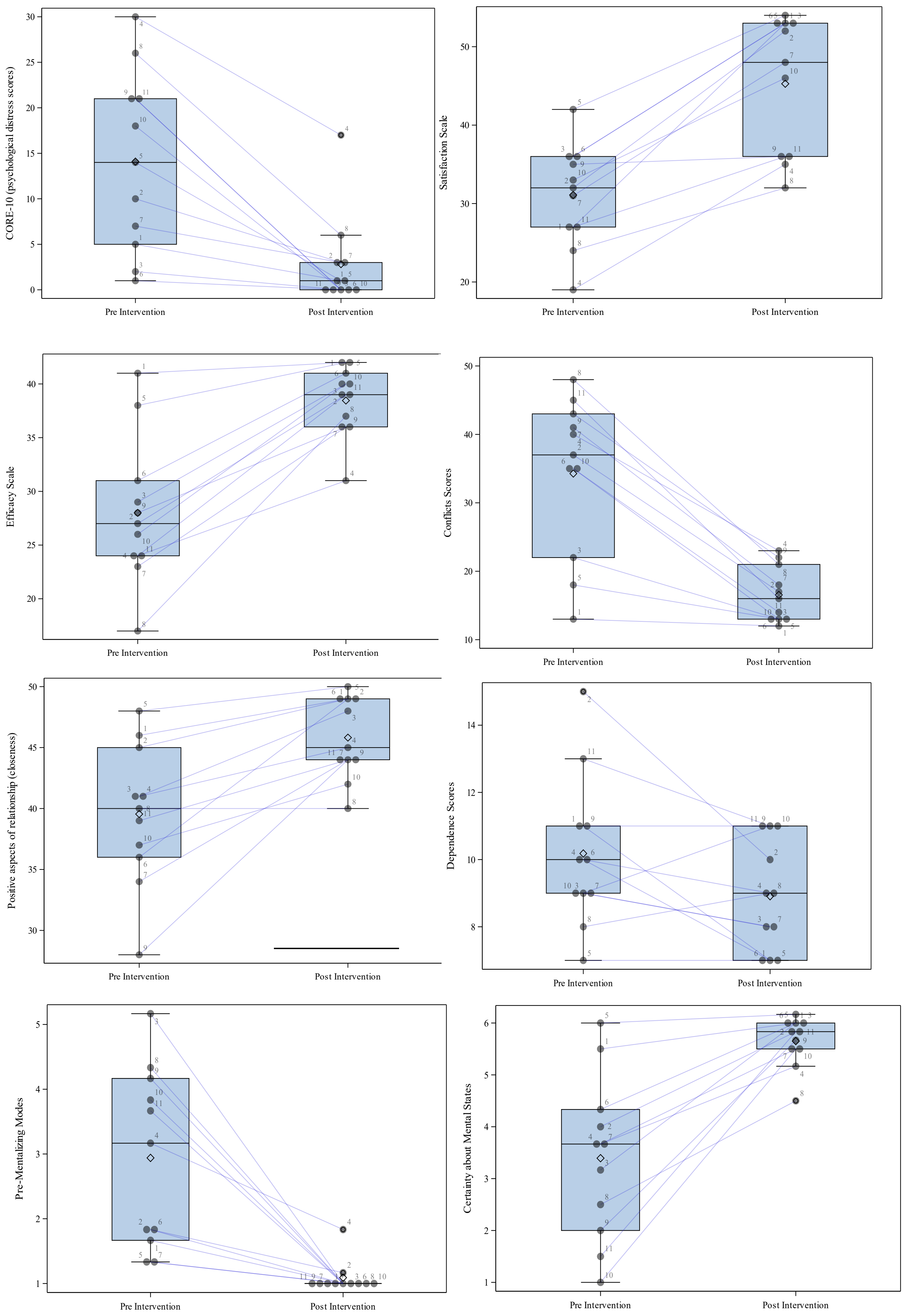

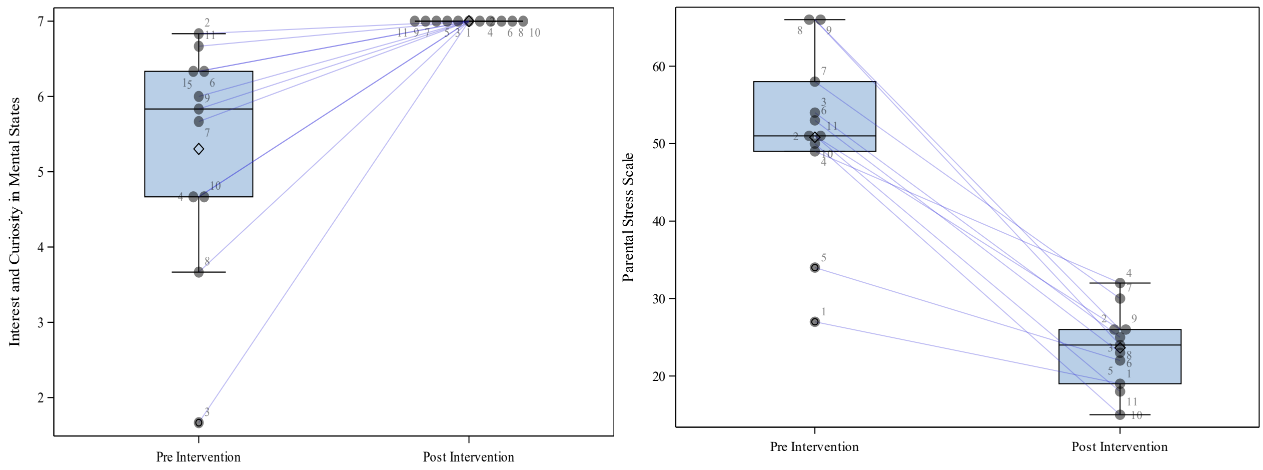

Figure 1. *Graphs from right to left are CORE-10 psychological distress scores pre-and post-intervention; Being a parent questionnaire (Satisfaction subscale) scores pre-and post-intervention; Being a parent questionnaire (Efficacy subscale) scores pre-and post-intervention; Child-Parent Relationship (Conflict subscale) scores pre-and post-intervention; Child-Parent Relationship Scale (Closeness subscale) scores pre-and post-intervention; Child-Parent Relationship Scale (Dependence subscale) scores pre-and post-intervention; The parental reflective functioning (pre-mentalizing modes) pre-and post-intervention; The parental reflective functioning scale (certainty about mental states) pre-and post-intervention; The parental reflective functioning scale (interest and curiosity in mental states) pre-and post-intervention and; The parental stress scale scores pre-and post-intervention.*
